# Effect of a heated humidifier during continuous positive airway pressure delivered by a helmet

**DOI:** 10.1186/cc6875

**Published:** 2008-04-21

**Authors:** Davide Chiumello, Monica Chierichetti, Federica Tallarini, Paola Cozzi, Massimo Cressoni, Federico Polli, Riccardo Colombo, Antonio Castelli, Luciano Gattinoni

**Affiliations:** 1 Unità Operativa di Anestesia e Rianimazione, Fondazione IRCCS – 'Ospedale Maggiore Policlinico, Mangiagalli, Regina Elena', via F. Sforza 35, 20122 Milan, Italy; 2 Istituto di Anestesiologia e Rianimazione, Università degli Studi di Milano, via F. Sforza 35, 20122 Milan, Italy; 3 Unità Operativa di Anestesia e Rianimazione, Ospedale Luigi Sacco, via G.B. Grassi, 20157 Milan, Italy

## Abstract

**Introduction:**

The helmet may be an effective interface for the delivery of noninvasive positive pressure ventilation. The high internal gas volume of the helmet can act as a 'mixing chamber', in which the humidity of the patient's expired alveolar gases increases the humidity of the dry medical gases, thus avoiding the need for active humidification. We evaluated the temperature and humidity of respiratory gases inside the helmet, with and without a heated humidifier, during continuous positive airway pressure (CPAP) delivered with a helmet.

**Methods:**

Nine patients with acute respiratory failure (arterial oxygen tension/fractional inspired oxygen ratio 209 ± 52 mmHg) and 10 healthy individuals were subjected to CPAP. The CPAP was delivered either through a mechanical ventilator or by continuous low (40 l/min) or high flow (80 l/min). Humidity was measured inside the helmet using a capacitive hygrometer. The level of patient comfort was evaluated using a continuous scale.

**Results:**

In patients with acute respiratory failure, the heated humidifier significantly increased the absolute humidity from 18.4 ± 5.5 mgH_2_O/l to 34.1 ± 2.8 mgH_2_O/l during ventilator CPAP, from 11.4 ± 4.8 mgH_2_O/l to 33.9 ± 1.9 mgH_2_O/l during continuous low-flow CPAP, and from 6.4 ± 1.8 mgH_2_O/l to 24.2 ± 5.4 mgH_2_O/l during continuous high-flow CPAP. Without the heated humidifier, the absolute humidity was significantly higher with ventilator CPAP than with continuous low-flow and high-flow CPAP. The level of comfort was similar for all the three modes of ventilation and with or without the heated humidifier. The findings in healthy individuals were similar to those in the patients with acute respiratory failure.

**Conclusion:**

The fresh gas flowing through the helmet with continuous flow CPAP systems limited the possibility to increase the humidity. We suggest that a heated humidifier should be employed with continuous flow CPAP systems.

## Introduction

During normal spontaneous breathing, ambient air – apart from being filtered for particles and micro-organisms by the nose and upper airways – is heated to body temperature and humidified, so that it is saturated by the time it reaches the alveoli [1]. Consequently, when the upper airways are bypassed (as in a patient with an endotracheal tube) medical gases, which are drier than ambient air [2], must be heated and humidified by heated humidifiers or heat-moisture exchangers [1] in order to avoid bronchial inflammation, cell damage, impairment of mucociliary clearance and loss of pulmonary function [3-6].

During noninvasive positive pressure ventilation (NPPV; defined as any form of ventilation support applied without an endotracheal tube [7]) the upper airways are not bypassed, and so theoretically they should adequately heat and humidify medical gases. However, out of a group of patients with severe obstructive sleep apnoea syndrome who were treated with nasal continuous positive airway pressure (CPAP) and administered nonhumidified ambient air, up to 60% suffered from nasal congestion, stuffiness and dryness [8,9]. These airway symptoms were mainly caused by the considerable one-way flow of ambient air through the nose and out of the mouth during mouth leaks, causing the release of several vasoactive amines and leukotrienes [10,11]. The use of a heated humidifier reduced nasal congestion [10,12], upper airway dryness, and dry mouth or nose [13]. In addition, the use of a heated humidifier increased patient satisfaction and the number of hours with CPAP application per night, and the patients indicated that they felt more refreshed on awakening [14].

In clinical practice, the use of a heated humidifier during CPAP is regarded to be optional, and it is normally utilized only when the patient exhibits symptoms of nasal or oral dryness or during prolonged utilization [15-19]. A recent review of NPPV [20] concluded that humidification is usually unnecessary during short-term application of CPAP. However, the last international consensus conference on NPPV in intensive care [7] stated that inadequate humidification of medical gases may cause patient distress, especially if the gases are supplied via a pipeline or cylinder.

At the present time there is no information on the optimal level of humidity of inspired gases during the NPPV. The American National Standards Institute suggested, although not directly for NPPV, that 10 mgH_2_O/l of absolute humidity is the lowest acceptable level needed to minimize mucosal damage in the upper airways [21].

In the acute setting, NPPV is usually delivered via a face mask, but this may cause discomfort, skin lesions and gas leaks [7]. To enhance patient comfort and to permit longer periods of NPPV, a new device – the 'helmet' – has been introduced [22-24]. Similar to the carbon dioxide rebreathing that occurs with use of the helmet [25], the high internal gas volume could also serve as a 'mixing chamber' between the heated and humidified expired gases and the dry medical gases entering the helmet. This could raise the levels of heat and humidity of the medical gases, thus avoiding the need for a heated humidifier. The final humidity inside the helmet will depend mainly on two factors: the amount of humidity in the patient's expired gases and the flow of fresh medical gases into the helmet. In addition, the humidifying capability of the respiratory tract may also be influenced by the presence of airway or pulmonary disease [26-28].

The aim of this study, conducted in patients with acute respiratory failure and in a group of healthy individuals, was to evaluate the temperature and humidity of respiratory gases within the helmet, with and without a heated humidifier, during CPAP delivered with a continuous high-flow and low-flow system or with a modern mechanical ventilator.

## Materials and methods

### Population

Two groups of individuals were studied. The first group included nine patients with acute respiratory failure who required CPAP. The patients' characteristics are shown in Table 1. To be eligible for inclusion in the study, the patients were required to be clinically and haemodynamically stable. The second group included 10 healthy nonsmokers (four males, mean age 25.5 ± 2.8 years and weight 74.8 ± 16.1 kg) with no airway disease, rhinitis, nasal surgery, or upper airway infections during the preceding month.

**Table 1:** Patients' characteristics

Patient	Age (years)	Sex	Weight (kg)	CPAP (cmH_2_O)	PaO_2_/FiO_2 _(mmHg)	PaCO_2 _(mmHg)	pH	RR (breaths/minute)	Cause of ARF
1	51	Female	110	7.5	290	70	7.25	25	Acute exacerbation of COPD
2	78	Male	90	7.5	274	63	7.28	22	Acute exacerbation of COPD
3	63	Male	80	5	230	65	7.27	30	Acute exacerbation of COPD
4	77	Female	70	5	170	29	7.44	25	CHF
5	64	Male	95	5	216	37	7.38	25	Pneumonia
6	74	Female	75	5	170	38	7.45	24	Pneumonia
7	47	Male	90	10	155	34	7.51	25	Pneumonia
8	79	Female	70	10	142	39	7.45	20	CHF
9	72	Female	70	10	225	41	7.47	25	CHF

Mean ± SD	67.2 ± 11.8	4 males/5 females	83.3 ± 13.9	7.2 ± 2.3	209 ± 52	46.2 ± 15.3	7.39 ± 0.1	24.5 ± 2.7	

ARF, acute respiratory failure; CHF, congestive heart failure; COPD, chronic obstructive pulmonary disease; CPAP, continuous positive airway pressure; FiO_2_, inspired oxygen fraction; PaO_2_, partial arterial pressure of oxygen; RR, respiratory rate; SD, standard deviation.

Each patient was informed about the procedure and a physician not involved in the study protocol was present to provide patient care. The study was approved by the institutional review board of our hospital and informed consent was obtained in accordance with Italian national regulations.

### Interface

The helmet (Castar, Starmed, Modena, Italy) is a transparent, latex-free, polyvinylchloride hood, which is joined by a metal ring to a soft polyvinylchloride collar. Two underarm straps attached to the ring prevent it from moving upward when the gas pressurizes it.

The helmet has an internal gas volume of 15 l, which is reduced to approximately 12 l when the head is inserted into the helmet [24]. A physician excluded the possibility of air leakage by passing a hand around the collar of the helmet.

### Protocol

The individuals were studied in the semirecumbent position. In the patients, the inspired oxygen fraction and the level of CPAP were maintained constant at the levels previously selected by the attending physician, whereas in healthy individuals the inspired oxygen fraction and the level of CPAP were kept constant at 21% and 5 cmH_2_O, respectively.

Different modes of ventilation were evaluated. The first mode was ventilator CPAP, delivered by a SERVO 300C ventilator (Maquet, Solna, Sweden) set to CPAP with the flow trigger regulated at medium sensitivity. The bias flow rate for the flow trigger was set at 2 l/minute. The second mode was continuous low-flow CPAP (40 l/minute; CPAP flow generator; Harold, Milan, Italy) delivered by a valveless system equipped with a latex reservoir bag with a volume of 10 l at atmospheric pressure and 20 l at 20 cmH_2_O pressure (Harold) [29]. The third mode was continuous high-flow CPAP (80 l/minute), delivered by the same valveless system as above [29]. A spring-loaded mechanical positive end-expiratory pressure valve (Medivalv, Vital Signs, Totowa, N.Y., USA) was used.

A Fisher & Paykel MR 730 heated humidifier (Fisher & Paykel, Auckland, New Zeland) equipped with a standard plastic disposable circuit was used as a conditioning system. The medical gas was conditioned by flowing it through a plastic chamber (the humidifying chamber) across the surface of warmed sterile water (vaporization surface). The temperature of the inspiratory gas was monitored by a probe at the end of the inspiratory line and at the chamber outlet. Theoretically, with the MR 730 the water reservoir was heated until the inspiratory gas at the end of the inspiratory line reached the preset temperature [30]. The temperature level of the heated humidifier was set at 37°C.

Each mode of ventilation was evaluated, in random order, with and without the heated humidifier. A total of six conditions were tested.

### Measurements

Room temperature was constant at 20 to 22°C. To stabilize the system in each condition, individuals were ventilated for at least 20 minutes before any measurements were taken.

A capacitive hygrometer (Hygroclip, Rotronic, Switzerland) was used to measure temperature and relative humidity inside the helmet (range for relative humidity 5% to 99%). The system incorporates a layer of plastic polymer between two electrodes, which – depending on humidity – can absorb molecules of water. Changes in capacity are correlated to the relative humidity. This system has a very low dead time and good accuracy, with an error of 0°C and no variations with time. At the end of each measurement the tip of the capacitive hygrometer was dried to avoid any possible measurement error.

The temperature and relative humidity inside the helmet were previously found to be similar in the different positions within the helmet.

All signals were amplified, digitized and recorded at 0.5 Hz using a data acquisition software (Colligo, Elekton, Milan, Italy). In each condition, on average, three or four readings from the probe were computed. The absolute humidity was computed using the following equation: absolute humidity = relative humidity × (0.0387 × T^^2 ^- 0.6066 × T + 13.776), where T is the temperature (in °C). The respiratory rate and tidal volume were obtained using the ventilator sensor. The absence of any autotriggering phenomena was ensured.

### Subjective evaluation

At the end of each study condition, patient comfort was rated using a continuous scale. Participants were asked to score their response to the question 'How do you feel in the present condition?' by placing a mark on a continuous line (length 10 cm), ranging from 'worst' (0 cm), 'poor' (2.5 cm), 'sufficient' (5 cm), 'good' (7.5 cm), to 'best feeling' (10 cm) [24]. The participants were carefully instructed on how to use the scale before starting the protocol.

### Statistical analysis

Ten patients were estimated to be necessary to demonstrate a difference of approximately 3 mgH_2_O/l in absolute humidity at a statistical significance of 0.05 (two tailed) and a power of 0.80 with a two-way analysis of variance design. Results are expressed as mean ± standard deviation. A *P *value less than 0.05 was considered significant. We compared the three CPAP systems with and without the heated humidifier using two-way analysis of variance for repeated measures, followed – when appropriate – by the Holm-Sidak test.

Comparisons within the same CPAP system in patients with acute respiratory failure and in healthy individuals were conducted using a *t*-test. The statistical analysis was performed using Sigma Stat Software (SPSS, Chicago, IL, USA).

## Results

The temperature and humidity of ambient air, pipeline medical gases and medical gases leaving the mechanical ventilator are shown in Table 2.

**Table 2:** Temperature and humidity of ambient air and of the medical gas under various conditions

	Temperature (°C)	Absolute humidity (mgH_2_O/l)	Relative humidity (%)
Ambient air	20–22	9–12	50–60
Pipeline medical gases	20–21	3–4	18–25
Ventilator medical gases	22–26	3–4	16–19

### Patients with acute respiratory failure

During ventilator CPAP the mean tidal volume and respiratory rate were 0.67 ± 0.12 l and 18.0 ± 4.4 breaths/minute, respectively. Application of the heated humidifier during all CPAP modes tested significantly raised the temperature, and absolute and relative humidity compared with CPAP without the heated humidifier (Table 3). Temperature and absolute and relative humidity were significantly higher with ventilator CPAP with and without the heated humidifier compared with continuous high-flow CPAP and continuous low-flow CPAP (with the exception of temperature for low-flow CPAP; Table 3). Continuous low-flow CPAP exhibited a significantly higher temperature, absolute and relative humidity compared with continuous high-flow CPAP.

**Table 3:** Temperature and humidity of the medical gas with and without the heated humidifier in patients with acute respiratory failure

	Temperature (°C)	Absolute humidity (mgH_2_O/l)	Relative humidity (%)
CPAP_VENT _with HH	32.4 ± 1.4^a,c^	34.1 ± 2.8^a,b,c^	98.1 ± 1.8^a,c^
CPAP_VENT _without HH	29.5 ± 2.0^a^	18.4 ± 5.5^a,b^	61.4 ± 14.7^a,b^
CPAP_LF _with HH	32.3 ± 1.0^a,c^	33.9 ± 1.9^a,c^	98.0 ± 1.0^a,c^
CPAP_LF _without HH	28.5 ± 1.7^a^	11.4 ± 4.8^a^	40.4 ± 15.5^c^
CPAP_HF _with HH	29.4 ± 1.1^c^	24.2 ± 5.4^c^	82.3 ± 18.3^c^
CPAP_HF _without HH	27.4 ± 1.2	6.4 ± 1.8	24.6 ± 6.9

^a^*P *< 0.05 versus CPAP_HF_; ^b^*P *< 0.05 versus CPAP_LF_; ^c^*P *< 0.05 versus each mode of CPAP without HH. CPAP, continuous positive airway pressure; CPAP_VENT_, ventilator CPAP; CPAP_LF_, continuous low-flow CPAP; CPAP_HF_, continuous high-flow CPAP; HH, heated humidifier.

**Figure 1:**
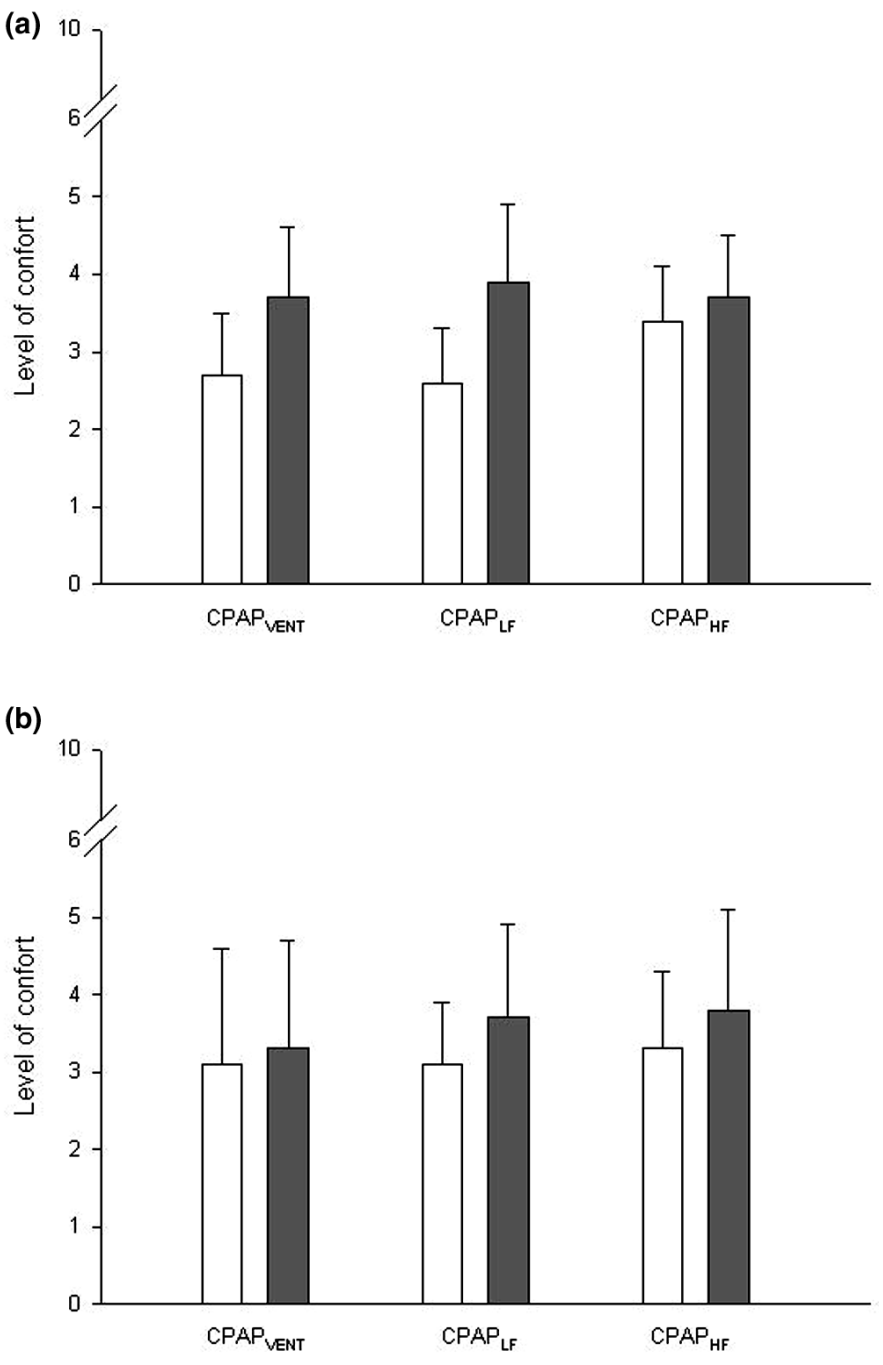
Comfort in patients with acute respiratory failure and healthy individuals with and without heated humidifier. Shown are average ratings of comfort in **(a) **patients with acute respiratory failure and in **(b) **healthy individuals with the heated humidifier (white bar) and without the heated humidifier (black bar). CPAP, continuous positive airway pressure; CPAP_HF_, continuous high-flow CPAP; CPAP_LF_, continuous low-flow CPAP; CPAP_VENT_, ventilator CPAP.

There was no difference in the ability to heat and humidify medical gases between the patients with acute respiratory failure and the healthy individuals. Level of comfort was quite good and without difference between the use or non-use of heated humidifier and the CPAP modes (Figure 1a).

### Healthy individuals

During ventilator CPAP, the mean tidal volume and respiratory rate were 1.02 ± 0.19 l and 12.2 ± 3.4 breaths/minute, respectively. The findings in healthy subjects and in patients with acute respiratory failure were similar except for the continuous low-flow and high-flow CPAP, which exhibited similar temperature and humidity (Table 4). Comfort level was similar with and without the heated humidifier and in all three CPAP modes (Figure 1b).

**Table 4:** Temperature and humidity of the medical gas with and without the heated humidifier in healthy individuals

	Temperature (°C)	Absolute humidity (mgH_2_O/l)	Relative humidity (%)
CPAP_VENT _with HH	31.7 ± 1.0^a,c^	32.8 ± 1.7^a,c^	98.1 ± 1.4^a,c^
CPAP_VENT _without HH	27.6 ± 1.3^a,b^	12.1 ± 3.4^a,b^	45.1 ± 10.9^a,b^
CPAP_LF _with HH	31.4 ± 0.5^a,c^	32.0 ± 0.8^a,c^	97.2 ± 1.6^a,c^
CPAP_LF _without HH	26.4 ± 1.3	7.4 ± 2.2	30.0 ± 8.4
CPAP_HF _with HH	28.5 ± 0.8^c^	24.6 ± 2.1^c^	88.3 ± 4.8^c^
CPAP_HF _without HH	25.9 ± 1.1	7.1 ± 1.1	29.8 ± 4.8

^a^*P *< 0.05 versus CPAP_HF_; ^b^*P *< 0.05 versus CPAP_LF_; ^c^*P *< 0.05 versus each mode of CPAP without HH. CPAP, continuous positive airway pressure; CPAP_VENT_, ventilator CPAP; CPAP_LF_, continuous low-flow CPAP; CPAP_HF_, continuous high-flow CPAP; HH, heated humidifier.

## Discussion

The main findings of this study, which evaluated the conditioning of medical gases during CPAP with helmet, were as follows. First, the use of a heated humidifier during ventilator CPAP, continuous low-flow and high-flow CPAP significantly increased the temperature and humidity of the gasses within the helmet. Second, taking 10 mgH_2_O/l as the absolute minimum humidity required for medical gases during NPPV, this level was achieved without use of the heated humidifier only during ventilator CPAP. Third, patients with acute respiratory failure and healthy individuals exhibited similar abilities to heat and humidify medical gases. Finally, use of the heated humidifier did not affect the level of patients' comfort.

The airway mucosa (nose, sinuses, trachea and bronchi) is highly vascular, rich in mucosal glands and covered by a hygroscopic mucus [1]. During spontaneous breathing, the ambient air, which under normal indoor conditions, has a temperature of 20°C to 24°C and an absolute humidity between 9 and 12 mgH_2_O/l, is progressively heated and humidified through the respiratory system by evaporation of water from the airway mucosa [31]. The air is heated and humidified until it becomes fully saturated by the time it reaches the alveoli (*i*.*e*., it reaches an absolute humidity of 44 mgH_2_O/l at a temperature of 37°C). During expiration, the temperature and humidity of the alveolar gas gradually drop, reaching a temperature of 32°C to 34°C and an absolute humidity of 27 to 34 mgH_2_O/l at the nose [1,32]. Thus, at least under normal ambient conditions, the temperature and humidity of the expired gases are always higher than those of ambient air [32,33].

Conversely, in patients with severe obstructive sleep apnoea receiving nasal CPAP delivering ambient air at high flow, the humidity of the inspired gas fell significantly [16]. This high flow of ambient air through the nose increased nasal resistance [12,13], causing discomfort as a result of dryness of the nose and mouth [10]. However, a heated humidifier, by increasing the humidity of the air, relieves or prevents these symptoms [13,14,16].

Medical gases, which are a mixture of oxygen with air, supplied through a pipeline or from a cylinder, are drier than ambient air (Table 2). When these gases are not adequately humidified they can deplete the moisture of the mucosa, reducing ciliary activity and causing functional alterations in the upper airway epithelium [1,3]. It appears reasonable from a physiological point of view that the medical gases should more closely mimic the conditions of normal ambient air [34].

In patients with acute respiratory failure, the face mask is more often used to deliver CPAP rather than a nasal mask because it can deliver higher ventilation pressures [7]. The face mask, with its higher anatomical dead space than that of the nasal mask, can maintain the humidity returned during the expiratory phase, thus increasing the humidity of the inspired gases and counterbalancing the difference between the dry inspired gases and the expired gases at each cycle. Araujo and coworkers [16] found similar levels of humidity using a face mask without a humidifier and a nasal mask with a humidifier. The helmet, which may be a valid alternative interface to the face mask during CPAP [22,24], has a high internal gas volume, similar to a semiclosed environment [25] in which the expired heat and humidity can mix with the fresh gas, thus raising the temperature and humidity of the dry medical gases.

The indications for NPPV depend on the goals of therapy, for instance to reduce the carbon dioxide or to improve oxygenation in patients with acute respiratory failure, and may differ depending upon the clinical context [7]. However, the absence of large-scale controlled studies obtained in different types of populations mean that NPPV cannot unequivocally be indicated in all patients with acute respiratory failure [7,20].

In the present study, we found higher humidity with ventilator CPAP than with the continuous flow CPAP system, and it was also higher than the lowest level required [21,34]. This suggests that the helmet acts as a 'humidity mixing chamber' between expired gases and dry medical gases, making it unnecessary to use a heated humidifier.

Continuous gas flow, as compared with the intermittent flow that occurs during ventilator CPAP, dilutes expired humidity to a greater extent, and therefore it is more difficult to humidify the medical gases adequately. In these conditions a heated humidifier is necessary. Any leakage from the helmet during ventilator CPAP could increase the gas flow delivered by the ventilator to maintain the positive end-expiratory pressure level constant, causing an increase in the proportion of the dry fresh gas mixing with the expired gases, thus reducing the ability to achieve adequate humidification.

The heated humidifier significantly increased the temperature and humidity of the medical gases, both by heating and humidifying the medical gases passing through the humidifying chamber, and by achieving a higher level of humidity in the expired gases as they mix with the gases inside the helmet [35]. The heated humidifier with the ventilator CPAP and the continuous low-flow CPAP caused a temperature and absolute humidity similar to those commonly employed during invasive mechanical ventilation [36]. However, with the continuous flow CPAP system, as opposed to the intermittent flow of ventilator CPAP, the heated humidifier did not deliver enough energy to heat the water in the humidification chamber properly, so the medical gases were less conditioned [30].

We studied a rather heterogeneous population of patients with acute respiratory failure, at a range of CPAP from 5 to 10 cmH_2_O. The main determinant of the temperature and humidity reached inside the helmet was the inspiratory gas flow passing through the helmet and not the level of CPAP.

In the present study the high temperature and absolute humidity reached using the heated humidifier with ventilator CPAP and continuous low-flow CPAP reduced the transparency of the helmet wall. However, patients rated their comfort similarly, independent of the level of humidity, at least for the short period of investigation reported here.

Previous studies have speculated that airway or pulmonary disease could interfere with the humidifying function of the respiratory tract [26-28]. We did not identify any difference between patients with acute respiratory failure and healthy individuals in terms of the temperature and humidity reached by the medical gases. This suggest (although it was not measured) that the humidity of the expired gases mixed with the fresh gases was similar in patients with acute respiratory failure and healthy individuals. Primiano and colleagues [33] found no difference in the temperature and humidity of the expired gases between patients with cystic fibrosis and healthy individuals breathing ambient air.

### Limitations

Possible limitations of this study must be clarified. First, although the level of humidity during ventilator CPAP was higher than the required minimum, we have no data on longer term use. Global patient comfort, without distinguishing noise, claustrophobia or sensation of heat, was also evaluated only over a short period. Second, only one type of helmet was used, with an internal volume of 15 l. However, similar to the case of 'carbon dioxide rebreathing' [25], the volume of the helmet should not directly influence the final level of humidity of the medical gases but only the rate at which the level is reached. We did not also measure leakages from the helmet during the intermittent gas flow delivered by the ventilator. By increasing the gas flow through the helmet, these leakages could result in greater dilution of heat and humidity of expired gases, reducing the temperature and humidity of the inspired gases. Third, because the overall duration of the study was no longer than 2 hours, this prevented us from evaluating other ventilatory modes. However, the data we obtained during ventilator CPAP (intermittent flow) could be also obtained during volume-controlled or pressure-controlled modes.

## Conclusion

During ventilator CPAP without a heated humidifier, the use of a helmet – acting as a mixing chamber between expired and inspired medical gases – increased the humidity of the inspired dry gases to a degree similar to that of ambient air. However, the level of humidity reached with continuous flow CPAP systems, was lower than that of ambient air, and in these cases a heated humidifier is probably indicated.

## Key messages

• The helmet acts as a humidity chamber, rendering the use of a heated humidifier unnecessary during ventilator CPAP.

• Patients with acute respiratory failure and healthy individuals exhibit similar ability to heat and humidify medical gases.

• Use of the heated humidifier during CPAP does not affect patient comfort.

## Abbreviations

CPAP = continuous positive airway pressure; NPPV = noninvasive positive pressure ventilation.

## Competing interests

The authors declare that they have no competing interests.

## Authors' contributions

DC conceived of the study, participated in its design and coordination, performed the measurements and wrote a first draft of the manuscript. MC participated in the study design and coordination, performed the measurements and helped to draft the manuscript. FT participated in the study design and coordination, performed the measurements, and helped to draft the manuscript. PC participated in the study design and coordination, and performed the measurements. MC participated in the study design and coordination, and performed the measurements. FP participated in the study design and coordination, and performed the measurements. RC participated in the study design and coordination, and performed the measurements. AC participated in the study design and coordination, and performed the measurements. LG conceived of the study, participated in its design and coordination, coordinated the final analysis of collected data and revised the manuscript, writing its final version.
